# Complete mitochondrial genome of *Tolypocladium* sp. YFCC 1805002 isolated from *Ophiocordyceps sinensis* in Baima Snow Mountain, Southwestern China

**DOI:** 10.1080/23802359.2019.1698338

**Published:** 2019-12-13

**Authors:** Yanfang Liu, Dexiang Tang, Guodong Zhang, Mingxi Zhang, Yao Wang, Yuanbing Wang, Hong Yu

**Affiliations:** a Yunnan Herbal Laboratory, School of Life Sciences, Yunnan University, Kunming, China;; b The International Joint Research Center for Sustainable Utilization of Cordyceps Bioresources in China and Southeast Asia, Yunnan University, Kunming, China;; c The Research Center of Cordyceps Development and Utilization of Kunming, Yunnan Herbal Biotech Co. Ltd, Kunming, China

**Keywords:** Mitochondrial genome, phylogenetic analysis, *Tolypocladium*

## Abstract

*Tolypocladium* sp. YFCC 1805002, an undescribed species isolated from natural *Ophiocordyces sinensis* in Baima Snow Mountain, Deqin County of Yunnan in Southwestern China, was sequenced on the Illumina sequencing platform. The complete mitochondrial genome of this fungus is a circular molecule of 46,466 bp in length, and this value is higher than its two relatives *T. inflatum* and *T. ophioglossoides*. The circular mitogenome contains 15 protein-coding genes (PCGs), a set of 25 transfer RNA (tRNA) genes, and 2 ribosomal RNA (rns and rnl) genes. The 15 protein-coding genes are *atp6*, *8–9*, *cob*, *cox1–3*, *nad1–6*, *nad4L* and *rps3*. The lengths of 25 transfer RNA (tRNA) genes are ranging from 71 to 87 bp, and the sizes of *rns* and *rnl* are 1554 bp and 5931 bp, respectively. The overall base composition is 38.3% A, 35.6% T, 11.6% C, 14.5% G, with a low GC content of 26.1%. Phylogenetic analysis inferred from concatenated protein-coding genes of 51 taxa shows that the new species *Tolypocladium* sp. YFCC 1805002 is closely related to *T. inflatum* in the family Ophiocordycipitaceae with high credible support by Bayesian inference posterior probabilities (BI-PP = 100%). This study would facilitate the future research of genetics, evolution and medicine of cordycipitoid fungi.

The cordycipitoid genus *Tolypocladium* (Ophiocordycipitaceae, Hypocreales) was erected by the type species *T. inflatium*. Several species of *Tolypocladium* are medicinally and economically important due to the production of Cyclosporin A with antifungal activity and as immunosuppressant drug (Samson and Soares 1984; Li 1988; Bushley et al. 2013; Quandt et al. 2015, Rocha et al. 2015; Yang et al. 2018; Zhang et al. 2018). Due to the importance of *Tolypocladium* for mycology, medicine and ecology, more research is necessary to obtain genomic information of this lineage. Based on morphological and nuclear gene phylogenetic evidence, a fungal strain YFCC 1805002 isolated from the natural *Ophiocordyceps sinensis* specimen was identified as a new species of *Tolypocladium* and would be described elsewhere. This study aims to report the complete mitochondrial genome (mitogenome) of *Tolypocladium* sp. YFCC 1805002 and to decipher its phylogenetic relationship to other cordycipitoid fungi.

*Tolypocladium* sp. YFCC 1805002 was isolated from the natural fresh *O. sinensis* specimen collected from Baima Snow Mountain, Deqin County of Yunnan in Southwestern China (28°26′02″N, 99°01′33″E, alt. 4902 m). The strain was deposited at the Yunnan Fungal Culture Collection (YFCC), Yunnan University. Mycelia cultured on PDA at 20 °C for 25 days without light condition were prepared to extract total genomic DNA using DNeasy Plant Genomic DNA Purification Mini Kit (QIAGEN). The whole-genome sequencing was carried out by Novogene Co., Ltd. (Beijing, China) on the Illumina sequencing platform (HiSeq-PE150). The software SPAdes v. 3.11.0 was used to assemble mitogenome of *Tolypocladium* sp. YFCC 1805002 (Bankevich et al. 2012). Mitogenome was annotated using MFannot tool and ARWEN web server, combined with artificial correction technology. To draw the mitogenomic circular map, the Organellar Genome DRAW tool was employed (Lohse et al. 2007).

The annotated mitogenome was submitted to GenBank under accession No. MN 583265. The total length of this circular mitogenome is 46,466 bp, which is higher than those of its two relatives, namely 25,328 bp of *T. inflatum* and 35,159 bp of *T. ophioglossoides*. The circular mitogenome contains 15 protein-coding genes (PCGs), a set of 25 transfer RNA (tRNA) genes and 2 ribosomal RNA (rns and rnl) genes. The 15 PCGs are *atp6*, *8–9*, *cob*, *cox1–3*, *nad1–6*, *nad4L* and *rps3*. The lengths of 25 tRNA genes are ranging from 71 to 87 bp, and the sizes of *rns* genes and *rnl* are 1554 bp and 5931 bp, respectively. The overall base composition is as follows: 38.3% A, 35.6% T, 11.6% C, 14.5% G, with a low GC content of 26.1%.

To dertermine the phylogenetic relationship of *Tolypocladium* sp. YFCC 1805002 and its allies, fourteen concatenated mitochondrial PCGs in total of 12,972 bp were employed for phylogenetic analysis. Sequence alignment and phylogenetic analysis were conducted as described by Wang et al. (2018). Phylogenetic tree showed that the new species *Tolypocladium* sp. YFCC 1805002 is clustered together with *T. inflatum* in the family Ophiocordycipitaceae with high credible support by Bayesian inference (BI) posterior probabilities (BI-PP = 100%), but showing the two has distant genetic distance (Figure 1).

**Figure 1.**
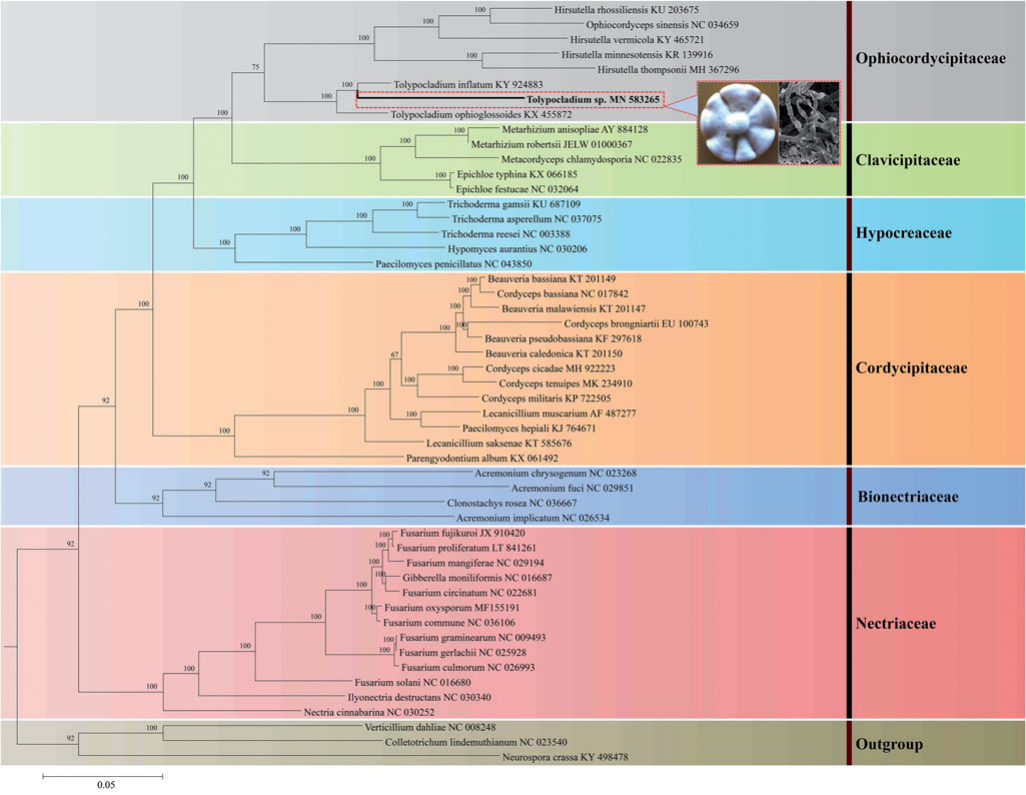
Phylogenetic relationships among 51 taxa of Sordariomycetes based on analysis from 14 concatenated mitochondrial protein-coding genes (PCGs). The 14 PCGs include subunits of the respiratory chain complexes (*cob, cox1, cox2, cox3*), ATPase subunits *(atp6, atp8, atp9*), NADH: quinone reductase subunits (*nad1, nad2, nad3, nad4, nad4L, nad5, nad6*). The phylogenetic tree is generated by Bayesian inference (BI) and posterior probabilities are shown above internodes. Colony and asexual conidiogenous structure of *Tolypocladium* sp. YFCC 1805002 are presented in the phylogenetic tree.
